# Roles and Mechanisms of Regulated Necrosis in Corneal Diseases: Progress and Perspectives

**DOI:** 10.1155/2022/2695212

**Published:** 2022-05-23

**Authors:** Wanying Lin, Minting Chen, Yacouba Cissé, Xiaofeng Chen, Lang Bai

**Affiliations:** Department of Ophthalmology, Nanfang Hospital, Southern Medical University, Guangzhou, China

## Abstract

Regulated necrosis is defined as cell death characterized by loss of the cell membrane integrity and release of the cytoplasmic content. It contributes to the development and progression of some diseases, including ischemic stroke injury, liver diseases, hypertension, and cancer. Various forms of regulated necrosis, particularly pyroptosis, necroptosis, and ferroptosis, have been implicated in the pathogenesis of corneal disease. Regulated necrosis of corneal cells enhances inflammatory reactions in the adjacent corneal tissues, leading to recurrence and aggravation of corneal disease. In this review, we summarize the molecular mechanisms of pyroptosis, necroptosis, and ferroptosis in corneal diseases and discuss the roles of regulated necrosis in inflammation regulation, tissue repair, and corneal disease outcomes.

## 1. Introduction

As an endpoint of the cell life cycle, cell death has a significant role in physiological processes, such as immunity, development, and tissue homeostasis [1]. According to The Nomenclature Committee on Cell Death, cell death is classified into two groups: accidental cell death and regulatory cell death [2]. Among them, regulatory cell death, also known as programmed cell death, is categorized as noninflammatory (apoptosis) and inflammatory cell death (regulated necrosis). Apoptosis is characterized by intact cell membrane, cell shrinkage, membrane blebbing, chromatin condensation, nuclear fragmentation, and apoptotic body formation, without the induction of inflammation. Compared to apoptosis, regulated necrosis is a genetically controlled cell death process that is characterized by organelle and cell swelling, disrupted cell membrane, cytoplasmic content release, and inflammatory responses [3, 4]. Appropriate inflammatory responses enhance the ability of the immune system to fight infections through leukocyte migration, while pathological inflammatory responses induced by regulated necrosis cause tissue injury and aggravate inflammatory responses [5].

Regulated necrosis has multiple forms including pyroptosis, necroptosis, ferroptosis, and parthanatos. These forms are driven by different molecular pathways. Pyroptosis is a gasdermin-mediated programmed necrotic cell death that involves inflammatory caspase activation and plasma membrane pore formation [6, 7]. Necroptosis, a form of caspase-independent cell death, is mainly regulated by receptor-interacting protein kinase 1 (RIPK1), receptor-interacting protein kinase 3 (RIPK3), and mixed-lineage kinase domain-like protein (MLKL) [8]. Ferroptosis can be activated by inhibition of glutathione peroxidase 4 (GPX4) (the membrane repair enzyme) or the glutamate/cystine antiporter (xCT), whereas it can be inhibited by iron chelators, lipophilic antioxidants, polyunsaturated fatty acid phospholipids (PUFA-PLs), and lipid peroxidation inhibitors [9, 10]. All these regulated necroses participate in the development of multiple diseases, including cardiocerebrovascular injury [11], neurological disease [12], ischemic stroke injury [4, 13], digestive diseases [14], kidney diseases [15–17], liver diseases [18], endocrine diseases [19], hypertension [20], pulmonary disease [21, 22], and cancer [7, 23, 24]. Moreover, it has also been implicated in the pathogenesis of corneal diseases. Further details on the features of regulated necrosis have been described previously by comprehensive review articles [25–28].

Corneal disease refers to a group of disorders that are caused by dysfunctional cornea and is one of the leading causes of blindness worldwide [29–31]. Various chemical, physical, and pathological insults induce corneal edema, opacity, ulceration, perforation, and neovascularization [32, 33]. Damage to the cornea impairs the barrier function of the cornea and causes refractive errors and visual loss. The currently available treatments for some corneal diseases include drugs (e.g., antibiotics) and surgeries (e.g., corneal transplantation). However, these treatments are not sufficiently effective and are associated with surgical complications which lead to poor prognostic outcomes and disease recurrence [34]. Therefore, elucidation of the mechanisms involved in corneal diseases will inform the development of novel therapeutic strategies. This review describes the molecular mechanisms of pyroptosis, necroptosis, and ferroptosis and their relevance to corneal disease development.

## 2. Relevance of Pyroptosis in Corneal Diseases

**Figure 1:**
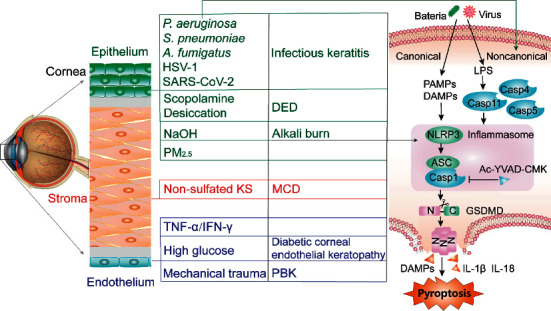
Schematic presentation of the potential contribution of pyroptosis to the death of corneal epithelial cells, corneal stromal cells, and corneal endothelial cells. SARS-CoV-2, severe acute respiratory syndrome coronavirus-2; DED, dry eye disease; KS, keratan sulfate; MCD, macular corneal dystrophy; PBK, pseudophakic bullous keratopathy; LPS, lipopolysaccharides; Casp, caspase; DAMPs, damage-associated molecular patterns.

In 2001, Cookson and Brennan termed pyroptosis as caspase-1-dependent nonapoptotic cell death [35]. Pyroptosis mechanisms are categorized into the caspase-1-dependent pathway (canonical pathway) and caspase-1-independent pathway (noncanonical pathway), both of which are driven by human caspase-4/5 or murine caspase-11 [23, 36]. In the canonical pyroptosis pathway, multiple pathogens and inflammatory agents, such as *Pseudomonas aeruginosa* (*P. aeruginosa*) and *Streptococcus pneumoniae* (*S. pneumoniae*), trigger the activation of the canonical NOD-like receptor pyrin 3 (NLRP3) inflammasome [37]. The NLRP3 inflammasome is an intracellular multiprotein complex that consists of the NLRP3 scaffold, adaptor protein ASC (apoptosis-associated speck-like protein containing a CARD), and caspase-1 [38]. Caspase-1 cleaves gasdermin *D* (GSDMD) and transforms the proinflammatory cytokine (pro-IL-1*β*) to generate mature IL-1*β* [38]. In the noncanonical pyroptosis pathway, oligomerization of caspase-4/5/11 activates the noncanonical NLRP3 inflammasome and triggers ASC recruitment, caspase-1 cleavage, and subsequent GSDMD cleavage [39]. Canonical and noncanonical pyroptosis are associated with GSDMD cleavage. The N-terminal fragment of GSDMD (GSDMD-NT) binds phospholipids on the plasma membrane and oligomerizes to form functional pores [40]. GSDMD pore formation causes mitochondrial dysfunction, cell ballooning, cell rupture, IL-1*β* release, and eventually the release of high mobility group 1 protein and ASC specks [36, 40]. GSDMD belongs to GSDMs, a family of intracellular proteins that are activated by proteases such as caspase-1/3/4/5/8/11 or granzyme A/B and execute pyroptosis [36]. Proteases also induce apoptosis, which can be switched to pyroptosis by high expression of GSDMs [36]. Both canonical and noncanonical pyroptosis are involved in the development of corneal diseases, including infectious keratitis [41–44], dry eye disease [45, 46], corneal alkali burn [47–49], macular corneal dystrophy [50], diabetic corneal endothelial keratopathy [51], and pseudophakic bullous keratopathy [52] (Figure 1).

### 2.1. Pyroptosis and Infectious Keratitis

In developed and developing countries, infectious keratitis is the most common cause of corneal blindness [32, 53]. In China, the prevalence of past or active infectious keratitis is 0.192% [54]. Based on its etiological agent, infectious keratitis can be classified as bacterial, fungal, or viral keratitis [32, 53]. Microorganisms induce excess and uncontrolled inflammation as well as corneal epithelial defects, resulting in corneal haze, ulcers, perforations, and vision loss [54]. Infectious keratitis is treated using topical applications of antibiotics (e.g., chloramphenicol and levofloxacin), antifungal (e.g., voriconazole, fluconazole, amphotericin B, and natamycin), and antiviral eye drops (e.g., acyclovir and derivatives) [44, 55, 56]. However, prolonged use of antibiotics, antifungal agents, and antiviral agents elicits resistance from microorganisms which has become a global problem [55, 57].

It is reported that microorganisms can induce pyroptosis in corneal epithelial cells. After infections, abnormal inflammasome activation leads to excess inflammation, which injures inflamed corneal tissues [44]. Moreover, caspase-1 levels are significantly elevated in *P. aeruginosa* [41], *Aspergillus fumigatus* (*A. fumigatus*) [43], and herpes simplex virus 1 (HSV-1) keratitis [44]. Given the significance of pyroptosis in infectious keratitis, various classes of pyroptosis inhibitors, including caspase-1 inhibitors, have been developed to therapeutically target pyroptosis.

#### 2.1.1. Bacterial Keratitis

Bacterial infections can lead to corneal perforation or blindness. Studies from Taiwan and Iran have shown that *P. aeruginosa* is the most common pathogen in young patients with bacterial keratitis, while *S. pneumoniae* is the most prevalent pathogen in elderly patients [55]. Clinically, *P. aeruginosa* and *S. pneumoniae* keratitis progress rapidly, resulting in corneal injury, including corneal epithelial defects, ulcers, suppurative infiltration of the stroma, neovascularization, perforation, and vision loss [58, 59].

*P. aeruginosa* and *S. pneumoniae* infections have been reported to exacerbate keratitis through the canonical pyroptosis pathway. In human corneal epithelial cells or mice keratitis models, *P. aeruginosa* upregulated the inflammasome as well as pyroptosis-associated genes and activated the NLRP3/caspase-1/IL-1*β* pathway [41, 42]. The caspase-1 inhibitor, Ac-YVAD-CMK, blocked pyroptosis and relieved the symptoms of *P. aeruginosa* keratitis, suggesting that caspase-1 is a potential target for bacterial keratitis therapy [41]. In mice models of *S. pneumoniae* keratitis, the NLRP3 inflammasome (NLRP3, ASC, and caspase-1) was proven to be essential for cleavage and secretion of corneal neutrophil-derived IL-1*β* and bacterial clearance [59]. These studies suggest that *P. aeruginosa* and *S. pneumoniae* induce corneal inflammasome activation, leading to canonical pyroptosis of corneal epithelial cells or tissues through the NLRP3/ASC/caspase-1/GSDMD/IL-1*β* axis.

Apart from canonical pyroptosis, noncanonical pyroptosis is also involved in *P. aeruginosa* keratitis. In the corneas of rats and patients with keratitis, *P. aeruginosa* enhanced the levels of caspase-4/5/11, cleaved GSDMD, and proinflammatory cytokine, which was rescued by wedelolactone [58]. Therefore, canonical and noncanonical pyroptosis contribute to bacterial keratitis development, which might be a new target for treating bacterial keratitis.

#### 2.1.2. Fungal Keratitis

Fungal keratitis accounts for over 50% of culture-positive microbial keratitis and is associated with agricultural trauma, especially in developing countries [43, 60]. *A. fumigatus* has been shown to induce corneal pyroptosis both *in vivo* and *in vitro*. The expression of GSDMD was significantly elevated in human corneal epithelial cells and mice models with *A. fumigatus* keratitis [43]. Pretreatment with GSDMD siRNA via subconjunctival injection alleviated keratitis by suppressing IL-1*β* levels as well as neutrophil and macrophage recruitment in mice models of *A. fumigatus* keratitis [43]. Moreover, GSDMD levels were inhibited by belnacasan (caspase-1 inhibitor) in human corneal epithelial cells infected with *A. fumigatus*, suggesting that GSDMD-mediated pyroptosis is associated with caspase-1 [43]. Based on these findings, GSDMD and caspase-1 are potential novel therapeutic targets for *A. fumigatus* keratitis [43].

In the pathological process of *A. fumigatus* keratitis, pyroptosis can also be regulated by upstream signals such as pannexin 1 and thymic stromal lymphopoietin. In human corneal epithelial cells and mice models of *A. fumigatus* keratitis, pannexin 1 channels contributed to IL-1*β* expressions via NLRP3/caspase-1 inflammasome [60]. In addition, thymic stromal lymphopoietin was secreted by human corneal epithelial cells upon *A. fumigatus* infection, which induces caspase-1-dependent pyroptosis and IL-1*β*/IL-18 secretion of macrophages through NLRP3 inflammasome activation [61]. Therefore, the pyroptosis signaling pathway and its upstream signals are involved in *A. fumigatus* keratitis progression.

#### 2.1.3. Viral Keratitis

HSV-1 and severe acute respiratory syndrome coronavirus-2 (SARS-CoV-2) can trigger pyroptosis in corneal epithelial cells. HSV-1 infects more than 52% of the world's population and is associated with minor epithelial herpes keratitis or blinding recurrent herpetic stromal keratitis [44]. HSV-1 in an infected cornea initiates virus replication in the corneal epithelial cells and stimulates the production of inflammatory cells, cytokines, and chemokines that gradually infiltrate into the stroma [62]. In human corneal epithelial cells or murine cornea, virulent strains of HSV-1 induced NLRP3, NLRP12, and IFI16 inflammasomes, activated caspase-1, and stimulated the secretion of inflammatory cytokines (IL-1*β* and IL-18), which resulted in neutrophil and inflammatory macrophage recruitment into the inflamed cornea [44].

The cornea may be a mode of transmission of SARS-CoV-2 [63]. It is reported that patients with coronavirus disease 2019 (COVID-19) may suffer from conjunctivitis and keratitis [64]. In the study by Zhu et al., stimulation of SARS-CoV-2 spike protein elevated GSDMD and IL-1*β* in human corneal epithelial cells, suggesting that SARS-CoV-2 induced pyroptosis [65].

### 2.2. Pyroptosis and Dry Eye Disease

Dry eye disease is a multifactor-induced autoimmune ocular surface disease that is characterized by an impaired balance between tear secretion, evaporation, and clearance [66]. In dry eye disease progression, the corneal epithelium can be affected by environmental factors. Environmental factors such as air pollutants, especially fine particulate matter (PM_2.5_), decrease tear film break-up time to induce corneal ulceration, epithelial defects, and neovascularization [67, 68]; desiccating stress and hyperosmolarity may lead to ocular damage and surface inflammation [69]. Activation of inflammatory mediators causes goblet cell loss, reduces mucus secretion, triggers epithelial cell apoptosis, and destroys tear film stability [69]. Blocking inflammatory mediators is beneficial for stabilizing tear films, tear production, and protection of the ocular surface.

Environmental factors and air pollutants can induce corneal pyroptosis to promote dry eye disease. The pyroptosis executor, N-GSDMD, was found to be elevated in dry eye patients' tears [45]. *In vivo*, desiccating stress-induced reactive oxygen species (ROS) generation triggered NLRP3-ASC-caspase-1 inflammasome formation and promoted IL-1*β* release in mice models of environment-induced dry eye disease, which was inhibited by N-acetyl-L-cysteine (ROS inhibitor) eye drops [46]. *In vitro*, hyperosmotic stress promoted the NLRP3/ASC/caspase-1/GSDMD pyroptosis pathway in human corneal epithelial cells, which was blocked by disulfiram and calcitriol [45]. Moreover, *in vivo* and *in vitro*, environmental stress-induced NLRP12/NLRC4 inflammasome activation mediated GSDMD cleavage to drive pyroptosis and IL-33 as well as IL-1*β* processing in corneal epithelium, which aggravated dry eye symptoms including tear loss, epithelial defects, and inflammatory responses [69]. Besides environmental factors, corneal PM_2.5_ exposure activates the NLRP3/ASC/caspase-1/GSDMD/IL-1*β*/IL-18 pyroptosis pathway, accompanied by increased ROS formation and decreased cell viabilities [70]. In summary, desiccating stress, hyperosmolarity, or PM_2.5_ initiates corneal epithelial pyroptosis through inflammasome-mediated and GSDMD-dependent signaling pathway in dry eye disease progression, suggesting that pyroptosis inhibitors are potential therapeutic options for dry eye disease.

### 2.3. Pyroptosis and Corneal Alkali Burn

Corneal alkali burn is an ophthalmic emergency and requires immediate diagnosis and prompt management [71]. Alkali burn suppresses corneal transparency through neovascularization and aggressive inflammatory response and even promote keratolysis, leading to globe perforation at the acute stage [72]. After corneal alkali burn, there are elevated levels of NLRP3, caspase-1, and IL-1*β* in corneal epithelial cells, along with corneal opacity and inflammation responses, which can be blocked by the NLRP3 inhibitor, butyrate, or pranoprofen [47, 48]. L-carnitine can also inhibit the NLRP3/caspase-1 pyroptosis pathway, promote the proliferation of stem/progenitor cells, and repair corneal epithelium after alkali burn [49]. Therefore, drugs targeting inflammasomes and pyroptosis can potentially treat corneal alkali burn.

### 2.4. Pyroptosis and Macular Corneal Dystrophy

Macular corneal dystrophy is an autosomal recessive disease that is caused by mutations in the carbohydrate sulfotransferase gene that affects keratan sulfate hydrophilicity [50, 73, 74]. Nonsulfated keratan sulfate has been reported to precipitate in the corneal stroma and causes corneal haze in macular corneal dystrophy patients [50]. Multiple signaling pathways including pyroptosis have been implicated in the pathogenic processes of macular corneal dystrophy [50]. In corneal stromal cells from the patients, an overload of nonsulfated keratan sulfate aggregations activated the NLRP3-caspase-1 inflammasome pathway and GSDME cleavage, leading to pyroptosis [50]. Administration of Ac-YVAD-CMK may prevent macular corneal dystrophy [50].

### 2.5. Pyroptosis and Corneal Endothelial Keratopathy

Loss of corneal endothelial cells resulted in corneal edema and vision loss in endothelial keratopathy, such as pseudophakic bullous keratopathy, diabetic corneal endothelial keratopathy, and Fuchs' endothelial corneal dystrophy [75]. Corneal endothelial cell pyroptosis participates in diabetic corneal endothelial keratopathy [51] and pseudophakic bullous keratopathy [52].

Diabetes can cause corneal endothelial keratopathy characterized by intercellular tight junction barrier damage and endothelial pump function disturbance in corneal endothelial cells [51]. The long noncoding RNA, KCNQ1OT1, was activated in the corneal endothelium of diabetic patients. KCNQ1OT1 induces pyroptosis through the KCNQ1OT1/miR-214/caspase-1 signaling pathway [51]. Caspase-1 transforms pro-IL-1*β* to generate mature IL-1*β*, which stimulates DNA infraction and pyroptosis in high glucose-treated human corneal endothelial cells and also exacerbates diabetic corneal endothelial keratopathy in diabetic rats [51].

Pseudophakic bullous keratopathy is caused by injuries to the corneal endothelial cell, including surgical mechanical trauma, intraocular infusions, and drug injection. In human corneal endothelial cells, exposure to tumor necrosis factor-*α* (TNF-*α*) and interferon-*γ* activated inflammasome and elevated oxidative stress, resulting in pyroptosis-induced cell loss [75]. Endothelial cell pyroptosis promotes inflammation and decreases the density of corneal endothelial cells, and these effects can be inhibited by Ac-YVAD-CMK [75]. In aqueous humor from pseudophakic bullous keratopathy patients, the protein levels of TNF*α*, interferon-*γ*, and ASC were also found to be significantly elevated [52, 76]. Moreover, ASC levels were positively correlated with central corneal thickness and severity of inflammation [52]. These results prove that the inflammasome is a promising therapeutic target for preventing loss of corneal endothelial cell.

## 3. Significance of Necroptosis in Corneal Diseases

**Figure 2:**
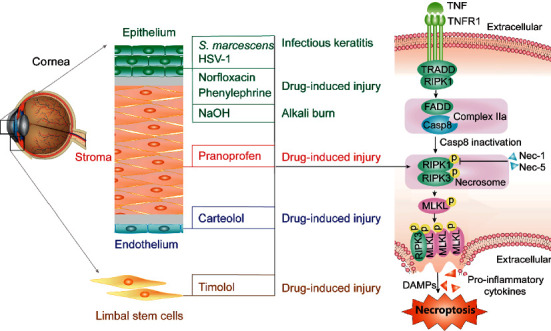
Schematic illustration of the potential contribution of necroptosis to the death of corneal epithelial cells, corneal stromal cells, and corneal endothelial cells. HSV-1, herpes simplex virus 1; TNFR1, TNF receptor 1; TRADD, TNF receptor-associated proteins with a death domain; FADD, Fas-associated death domain protein; Casp, caspase; Nec-1, necrostatin-1; Nec-5, necrostatin-5; DAMPs, damage-associated molecular patterns.

Degterev et al. first reported on necroptosis in 2005 [77]. It can be induced without caspase activation. Necroptosis is induced by various stimuli, such as TNF-*α*, tumor necrosis factor ligand superfamily member 6 (FasL), toll-like receptor 4, and interferon-*α*/*β* [78]. Once binding to the TNF receptor 1 on the cell membrane, TNF*α* forms the membrane-bound complex I. Complex I consists of TNF receptor-associated proteins with a death domain (TRADD), the Fas-associated death domain protein (FADD), the TNFR-associated factor 2, the cellular inhibitor of apoptosis protein 1/2 (cIAP1/2), and RIPK1 [14, 79]. After deubiquitination, RIPK1 interacts with FADD and caspase-8 to form complex IIa (FADD and caspase-8), which will, in turn, cause apoptosis [80]. When caspase-8 is inhibited, activated RIPK1 is ubiquitinated by Pellino-1 and binds RIPK3 to form complex IIb (necrosome), which phosphorylates MLKL and activates necroptosis, leading to membrane permeabilization and proinflammatory cytokines release. Necrostatin-1 can inhibit RIPK1 activation and prevents complex IIb formation to block necroptosis [27, 78]. Apart from caspase-8, caspase-2 is also a negative regulating factor of necroptosis. The RIPK1/3-mediated necroptosis is involved in pathophysiological processes of corneal diseases, including infectious keratitis [81, 82], corneal alkali burn, and drug-induced corneal injury (Figure 2).

### 3.1. Necroptosis and Infectious Keratitis

The mechanisms of bacteria-associated necroptosis have been extensively studied [83]. Necroptosis contributes to corneal epithelial cell death by dysregulating inflammation in infectious keratitis. *Serratia marcescens* (*S. marcescens*), a Gram-negative bacterium of the *Enterobacteriaceae family*, causes keratitis in neonates and immune-compromised patients as well as healthy individuals with contact lens [81]. In human corneal epithelial cells, *S. marcescens* induces cell membrane pore formation and necroptosis, which is rescued by RIPK1 and MLKL inhibitors (Necrostatin-5 and GW806742X) [53]. Therefore, necroptosis is a critical mechanism in corneal epithelial cell bubbles and death in *S. marcescens* keratitis [81].

Although necroptosis promotes *S. marcescens* infections, it also limits viral spread within the cornea and ganglia after HSV-1 infections. Guo et al. reported that seven days after corneal infection by HSV-1, tear films and trigeminal ganglia from RIPK3^−/−^ mice and Casp8^−/−^ RIPK3^−/−^ mice exhibited 10-fold higher amounts of infectious virus particles, relative to wild-type animals, suggesting that impairment of necroptosis and/or apoptosis enhances viral spread [82]. Moreover, the death rate of RIPK3^−/−^ mice and Casp8^−/−^RIPK3^−/−^mice was also higher than that of wild-type mice, implying that extrinsic apoptosis and necroptosis contribute to encephalitis resistance during HSV-1 acute infection [82]. Therefore, necroptosis promotes *S. marcescens* keratitis but protects against HSV-1 corneal infections. However, the reason for the diverse roles of necroptosis in bacterial and viral keratitis remains to be still elucidated.

### 3.2. Necroptosis and Corneal Alkali Burn

Corneal alkali burn can cause corneal opacity through corneal inflammation and neovascularization. The role of RIPK1 in ocular vascular disorders has been investigated. In mice models of corneal alkali burn, once-daily subconjunctival injections of necrostatin-1 prevented the increase in corneal neovascularization, and this beneficial effect was abrogated by combined injection of the caspase inhibitor, Z-VAD-FMK with necrostatin-1 [72]. This study reported the crucial role of RIPK1 in corneal neovascularization and suggested that disruption of apoptosis may trigger necroptosis [72]. This result is consistent with findings from previous studies, which provide evidence that cell death signaling skews towards necroptosis while blocking apoptosis [84].

### 3.3. Necroptosis and Drug-Induced Corneal Injury

Topical eye drops are widely used in the treatment of ophthalmic diseases. Among them, norfloxacin is an antibiotic that is clinically applied to treat keratitis. Phenylephrine is an alternative *α*1 receptor agonist that is used in mydriasis. Pranoprofen is commonly used to treat postoperative inflammation and pain being a nonsteroidal anti-inflammatory drug (NSAID) [85]. Carteolol and timolol are nonselective *β*-adrenoceptor antagonists for treating glaucoma [86, 87]. Clinical applications of these ocular drugs have been associated with corneal toxicity. Ocular drugs may lead to corneal cell death and decrease transparency [85]. There is a need to explore the cytotoxic mechanisms of ophthalmic drugs in corneal cells for rational drug use [88].

Ocular drugs tend to induce corneal cell apoptosis at low concentrations and necroptosis at high concentrations. *In vitro* or/and *in vivo*, low-dose norfloxacin (0.1875–0.75 mg/mL) [89] and phenylephrine (0.625%) [90] activated mitochondrion-dependent and caspase-mediated apoptosis of human corneal epithelial cells, whereas low-dose pranoprofen (0.00625%) [85], carteolol (0.015625–0.25%) [91], and medium-dose timolol (0.125%–0.0625%) [88], respectively, induced the apoptosis of human corneal stromal cells, human corneal endothelial cells, and rabbit limbal stem cells. As concentration increased, drug toxicity increased. At high doses (1.5–3.0 mg/mL norfloxacin, 10%–1.25% phenylephrine, 0.1%–0.0125% pranoprofen, 0.5–2% carteolol, and 0.25%–0.5% timolol), these ocular drugs activated the corresponding corneal cell necroptosis. Necroptotic corneal cells were characterized by nucleus swelling, chromatin condensation into small irregular patches, and morphological DNA diffusion, as well as activation of the RIPK1/RIPK3/MLKL/pMLKL cascade and inactivation of caspase-2/8 in signaling pathways. Besides ocular drugs, C6-ceramide, as the intermediate metabolite of sheath lipids in the cell membrane, also triggers necroptosis in human corneal stromal fibroblasts through autocrine production of TNF*α* and induction of the RIPK1/RIPK3 pathway [92]. The RIPK1 inhibitor, necrostatin-1, rescued human corneal endothelial cells from carteolol-induced necroptosis [91]. Therefore, the necroptosis inhibitor is a potential treatment option for drug-induced corneal injury.

## 4. Ferroptosis in Corneal Diseases

Ferroptosis, which was initially defined by Dixon in 2012, is a form of regulated necrosis [93]. Morphologically, ferroptosis is characterized by smaller than normal mitochondria with condensed membrane densities, reduced or vanished cristae, and ruptured outer membranes [94]. As an iron-dependent form of regulated necrosis, ferroptosis is characterized by three main events: cellular iron accumulation, glutathione depletion, and membrane lipid peroxidation [9, 95]. Various compounds, including erastin and RSL3, can induce ferroptosis, whereas the iron chelator (deferoxamine), lipophilic antioxidants (*α*-tocopherol, butylated hydroxytoluene, and *β*-carotene), and lipid peroxidation inhibitors can inhibit ferroptosis. Most upstream signals induce ferroptosis by inhibiting GPX4 or xCT (also named system *X*_*c*_^−^), followed by accumulation of lipid peroxidation and reduced glutathione biosynthesis [1]. The signaling pathways involved in ferroptosis include the xCT, GPX4, and Nrf2 pathways [10].

Evidence shows that xCT promotes intracellular glutathione synthesis via the glutamate-cystine antiporter system that exchanges glutamate out of the cell and cystine into the cell, which protects lipids, proteins, and DNA from oxidative stress [96]. Erastin and sulfasalazine induce ferroptosis by inhibiting xCT, resulting in glutathione biosynthesis reduction, lipid peroxidation, and ROS accumulation. Besides, the light chain subunit SLC7A11 of xCT can interact with the autophagy protein BECN1 to form a complex, which induces lipid peroxidation and ferroptosis.

GPX4 is a selenium-containing antioxidant enzyme that plays a unique role in reducing toxic lipid hydroperoxides [94]. Inactivation of GPX4 disrupts the clearance of intracellular peroxides and promotes ROS accumulation, leading to ferroptosis.

**Figure 3:**
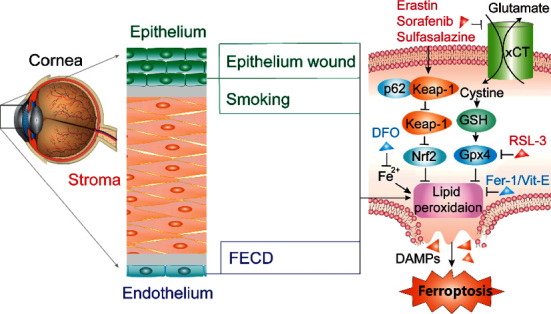
Schematic illustration of the potential contribution of ferroptosis to the death of corneal epithelial cells and corneal endothelial cells. Blue-colored words and red-colored words represent ferroptosis inhibitors and inducers, respectively. FECD, Fuchs' endothelial corneal dystrophy; DFO, deferoxamine; GSH, glutathione; Fer-1, ferrostatin-1; Vit-E, vitamin E; DAMPs, damage-associated molecular patterns.

Nuclear factor erythroid 2-related factor 2 (Nrf2) is an antioxidant regulator that inhibits ferroptosis [96]. Nrf2 is maintained at a low level under normal conditions and is degraded by the Kelch-like ECH-associated protein 1 (Keap1). Under oxidative stress, p62 expression suppresses Nrf2 degradation and promotes Nrf2 nuclear accumulation by inactivating Keap1 [96]. Additionally, activation of Nrf2-Keap1 signaling upregulates xCT and enhances glutamate secretion, thereby preventing cells from ferroptosis [97]. Ferroptosis plays an integral part in corneal diseases (Figure 3).

### 4.1. Ferroptosis and Corneal Epithelial Injury

GPX4 is an enzyme that regulates oxidative homeostasis, cell survival, and wound healing in corneal epithelial cells [98]. In GPX4^+/−^ mice models of n-heptanol-induced corneal epithelial injury, the loss of one GPX4 allele significantly delayed the healing of experimental corneal epithelial wounds [98]. In human corneal epithelial cells, cytotoxicity- and caspase-independent cell death-induced GPX4 deficiency was rescued by the ferroptosis inhibitor, *α*-tocopherol [98].

Smoking is a risk factor for corneal diseases, including dry eye disease [99]. In the human corneal epithelial cells, exposures to cigarette smoke extract or heated tobacco products activated the ferroptosis-signaling pathway via lipid peroxidation, ferritin cleavage, and ferrous ion accumulation, as well as cytokines IL-8 and IL-1*β* secretion. However, these effects were suppressed by either ferrostatin-1 or deferoxamine [99]. These findings show that ferroptosis is involved in corneal epithelial injury induced by n-heptanol, cigarette smoke extract, or heated tobacco products and that ferroptosis inhibitors can rescue the corneal epithelium from injury [98, 99].

### 4.2. Ferroptosis and Fuchs' Endothelial Corneal Dystrophy

Corneal endothelium death is permanent because it lacks regenerative capacities. Fuchs' endothelial corneal dystrophy is distinguished by suppressed corneal endothelial cell density and abnormal cell morphologies that lead to corneal edema and loss of vision [31, 100]. Suppression of Nrf2 in Fuchs' endothelial corneal dystrophy triggers a multitude of responses, including mitochondrial dysfunction, DNA damage, lipid peroxidation, and eventually, cell death [101]. In human corneal endothelial cells, Nrf2 loss induced a substantial increase in lipid peroxidation, causing ferroptosis, which was rescued by ferrostatin-1 [31]. Erastin/RSL3-induced ferroptosis in human corneal endothelial cells was rescued by complexed ubiquinol, which scavenges ROS and inhibits lipid peroxidation [102].

GPX4 participates in the maintenance of corneal redox state and protects corneal endothelial cells from oxidative stress. In human corneal endothelial cells, GPX4 knockdown significantly suppressed lipid peroxidation, inhibited cell proliferation, and enhanced hydrogen peroxide- and ferrous sulfate-induced cytotoxicity [103]. In conclusion, ferroptosis is involved in corneal endothelial keratopathy.

## 5. Summary

Pyroptosis, necroptosis, and ferroptosis play crucial roles in corneal diseases. This review described the significance of regulated necrosis in the death of corneal epithelial, stromal, and endothelial cells, which may be the main pathologic features for corneal diseases. This is important for identifying potential therapeutic targets for the treatment of corneal diseases.

Crosstalk has been shown to exist among regulated cell death pathways in corneal diseases. In animal models of corneal alkali burn, blocking apoptosis may activate necroptosis. Furthermore, different concentrations of ophthalmic drugs may induce corneal cell apoptosis and necroptosis. These findings imply that the crosstalk between corneal cell apoptosis and necroptosis is probably due to several molecular links among them. Although the significance of regulated necrosis in various corneal diseases has been widely investigated, the following question remains to be answered: Is one type of corneal disease regulated by different types of regulated necrosis? If the answer is yes, the underlying mechanisms and the temporal order, trigger, and crosstalk among these mechanisms in corneal disease should be determined.

In aqueous humor samples from pseudophakic bullous keratopathy patients, the levels of pyroptosis indicator, ASC protein, were found to be elevated, suggesting that indicators of the regulated necrosis pathway in aqueous humor or cornea can be used for early diagnosis. Furthermore, the intervention of the regulated necrosis pathway is a novel strategy for delaying or stopping corneal disease progression. In this review, we described the evidence supporting the hypothesis that targeting the pyroptosis, necroptosis, or ferroptosis pathways can reduce corneal epithelial, stromal, and endothelial cell deaths. For instance, the pyroptosis inhibitor, Ac-YVAD-CMK, rescued human corneal epithelial cells from cell death and relieved the symptoms of *P. aeruginosa* keratitis mice [41]. Necroptosis inhibitor, necrostatin-1, reduced corneal inflammation in mouse ocular surface and human corneal epithelial cells after exposure to particulate matter. This suggests that necrostatin-1 is a novel therapeutic target for the management of dry eye disease [104]. Ferroptosis inhibitors, ferrostatin-1 or deferoxamine, were found to suppress cell death and cytokines secretion in human corneal epithelial cell injury induced by either cigarette smoke [99]. To date, studies have investigated the benefits of targeting regulated necrosis as an effective approach to treating corneal disease using cellular and animal models, suggesting that local application of regulated necrosis inhibitors in the form of eye drops might be effective in treating corneal diseases. However, no clinical trials have been performed or are currently underway to test their effects and safety. Therefore, there is a long way to go before regulated necrosis inhibitors are suitable as a therapeutic option for clinical applications.

## Data Availability

The data supporting this review are from previously reported studies, which have been cited.
